# REVIEW: Emerging viral disease risk to pollinating insects: ecological, evolutionary and anthropogenic factors

**DOI:** 10.1111/1365-2664.12385

**Published:** 2015-01-16

**Authors:** Robyn Manley, Mike Boots, Lena Wilfert

**Affiliations:** ^1^ Centre for Ecology and Conservation University of Exeter Penryn Campus Penryn TR10 9EF UK

**Keywords:** pollinators, emerging disease, anthropogenic, biological risk factors, RNA viruses, transmission, infection, multihost pathogens, pollination, niche overlap

## Abstract

1. The potential for infectious pathogens to spillover and emerge from managed populations to wildlife communities is poorly understood, but ecological, evolutionary and anthropogenic factors are all likely to influence the initial exposure and subsequent infection, spread and impact of disease. Fast‐evolving RNA viruses, known to cause severe colony losses in managed honeybee populations, deserve particular attention for their propensity to jump between host species and thus threaten ecologically and economically important wild pollinator communities.
2. We review the literature on pollinator viruses to identify biological and anthropogenic drivers of disease emergence, highlight gaps in the literature, and discuss potential management strategies.
3. We provide evidence that many wild pollinator species are exposed to viruses from commercial species, resulting in multiple spillover events. However, it is not clear whether species become infected as a result of spillover or whether transmission is occurring within these wild populations. Ecological traits of pollinating insects, such as overlapping ranges, niches and behaviours, clearly promote cross‐species transmission of RNA viruses. Moreover, we conclude that the social behaviour and phylogenetic relatedness of social pollinators further facilitate within‐ and between‐host transmission, leaving these species particularly vulnerable to emerging diseases.
4. We argue that the commercial use of pollinators is a key driver of disease emergence in these beneficial insects and that this must be addressed by management and policy.
5. *Synthesis and applications*. There are important knowledge gaps, ranging from disease distribution and prevalence, to pathogen life history and virulence, to the impacts of disease emergence, which need to be addressed as research priorities. It is clear that avoiding anthropogenic pathogen spillover is crucial to preventing and managing disease emergence in pollinators, with far‐reaching effects on our food security, ecosystem services and biodiversity. We argue that it is crucial to prevent the introduction of diseased pollinators into natural environments, which can be achieved through improved monitoring and management practices.

## Abstract 2

There are important knowledge gaps, ranging from disease distribution and prevalence, to pathogen life history and virulence, to the impacts of disease emergence, which need to be addressed as research priorities. It is clear that avoiding anthropogenic pathogen spillover is crucial to preventing and managing disease emergence in pollinators, with far‐reaching effects on our food security, ecosystem services and biodiversity. We argue that it is crucial to prevent the introduction of diseased pollinators into natural environments, which can be achieved through improved monitoring and management practices.

## Introduction

Emerging infectious diseases can have devastating impacts on both managed and wild species (e.g. Strauss, White & Boots 2012) and indirectly threaten human welfare by depleting ecosystem services (Daszak, Cunningham & Hyatt 2000). Pathogen spillover from intensively managed populations poses a particular risk to susceptible wildlife communities that lack evolved resistance to novel pathogens (Daszak, Cunningham & Hyatt 2000; Colla *et al*. 2006). Pollinating insects are increasingly experiencing such viral disease spillover from managed honeybee (*Apis mellifera* and *A. cerana*) populations, and this has led to a burgeoning but disparate literature on disease occurrence in pollinators. Here, we review this literature to gain a better understanding of the various drivers of disease emergence, to highlight key knowledge gaps and to make management recommendations.

Insect pollinators are important for agriculture, food security and ecosystem function (Vanbergen *et al*. 2013), being responsible for the pollination of most flowering crops and wild plants (Klein *et al*. 2007). Indeed, Gallai *et al*. (2009) estimated the global value of insect pollinators at €153 billion per annum. Commercial pollination services are provided predominantly by honeybees *A. mellifera* and some bumblebee species, mainly *Bombus terrestris* (Europe and world‐wide), *B. impatiens* (North America) and *B. ignitus* (East Asia) (Velthuis & van Doorn 2006). In addition, wild pollinators play an important and often underestimated role in pollination of crops as well as native plants (Garibaldi *et al*. 2013). Yet, extinctions, reduced abundance and range contractions of wild and managed pollinator populations have been recorded in the Northern Hemisphere (reviewed by Vanbergen *et al*. 2013). Multiple interacting pressures, including habitat loss and fragmentation, agriculture intensification, climate change and emerging pathogens, are believed to be responsible for these recent declines (e.g. Vanbergen *et al*. 2013).

**Figure 1:**
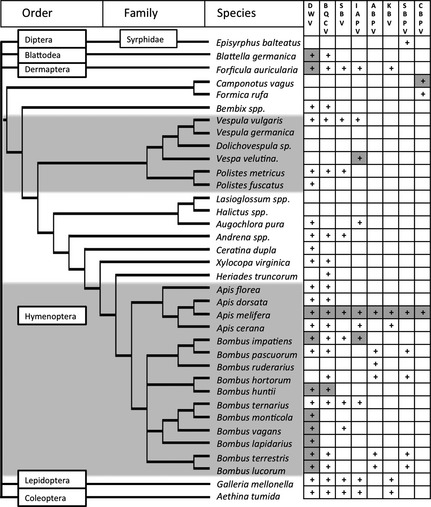
Phylogeny of pollinator species, and other insects associated with honeybee colonies, focussing on the Hymenoptera. Shaded species are social insects. ‘+’ indicates that the species has been identified as positive for virus, ‘
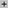
’ indicates virus replication has been demons trated. Virus abbreviations: DWV, deformed wing virus; BQCV, black queen cell virus; SBV, sacbrood virus; IAPV, Israeli acute paralysis virus; ABPV, acute bee paralysis virus; KBV, Kashmir bee virus; SBPV, slow bee paralysis virus; CBPV, chronic bee paralysis virus. Note that some data are based on small sample sizes, see Table S4.

Pathogens have emerged as a significant threat to the apicultural industry in recent years, with dramatic declines seen in populations of *A. mellifera*. While viral infections have been invoked as a potential cause of colony collapse syndrome (Cox‐Foster 2007; but see van Engelsdorp *et al*. 2009), the main culprit in pathogen‐related honeybee colony losses is infestation by the invasive mite *Varroa destructor*. This ectoparasite facilitates the spread of viral diseases and may increase their virulence (Martin 2001; Genersch 2010; Martin *et al*. 2012); see Table S1 in Supporting Information). In particular, one of these viruses (deformed wing virus, DWV) has recently been identified as an emerging disease in pollinators, with its prevalence in honeybees linked to its prevalence in wild bumblebees (Fürst *et al*. 2014). Although virological research has focused on honeybees, recent data suggest that many of the 24 viruses isolated from honeybees so far (de Miranda *et al*. 2013) have a broad host range, infecting some bumblebee, solitary bee, wasp, ant and hoverfly species (Fig. 1).

**Figure 2:**
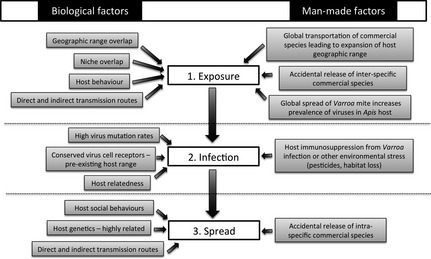
Identifying the main factors increasing the risk of RNA virus emergence in social pollinators.

Here, we review the potential for disease emergence within the pollinator community, based on data from the best‐studied honeybee RNA viruses [seven members of the Picornavirales; acute bee paralysis virus (ABPV), black queen cell virus (BQCV), DWV, Israeli acute paralysis virus (IAPV), Kashmir bee virus (KBV), sacbrood virus (SBV), slow bee paralysis virus (SBPV) and the unassigned chronic bee paralysis virus (CBPV)] (Table S2). We identify the biological and anthropogenic drivers that may promote successful disease emergence within the pollinator community, from (1) the initial exposure of the pathogen to novel hosts, to (2) successful infection in a novel host and, finally, (3) transmission within a novel host species (Fig. 2).

## Exposure of novel hosts to viruses

The first step in disease emergence is the exposure of a potential novel host to the pathogen. Both biological and anthropogenic drivers can influence the frequency and extent of contact between a reservoir and novel host population, thus increasing the risk of transmission and disease emergence.

### Biological drivers: transmission and distribution

#### Prevalence and geographic range of viruses

**Figure 3:**
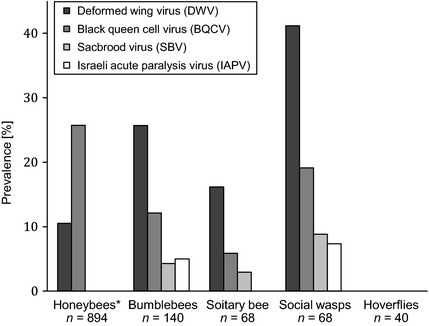
Cumulative percentage prevalence of DWV, BQCV, SBV and IAPV across pollinator species groups. *Note that ‘honeybees’ exclude *A. mellifera –* (data from Singh *et al*. 2010; Evison *et al*. 2012; Li *et al*. 2012; Zhang *et al*. 2012; Levitt *et al*. 2013), *n* = total number of individuals sampled within each species group. See Table S4 for a list of species and raw data.

High prevalence and large geographic range increase a pathogen's potential to encounter novel host species. Honeybees are now kept in most inhabited areas of the world, and many pathogens have accompanied their host in this global spread (Ellis & Munn 2005). Of the eight commonly studied viruses, most are reported globally (Table S1). Comparisons between studies are difficult because viral prevalence can vary between castes and through seasons (Chen & Siede 2007) and sampling effort and methods differ. Despite this, it is clear from available data that some viral pathogens (particularly DWV and BQCV) generally have high prevalence, infecting the majority of honeybee hives where they are present (Table S1). This high prevalence in honeybees is mirrored by the high DWV presence in other pollinator species surveyed in the USA and UK (Fig. 3). The near‐ubiquitous presence of honeybees and the generally high prevalence of both asymptomatic and pathogenic virus infections across apiaries provides ample opportunity for cross‐species transmission.

#### Mode of transmission

A pathogen's transmission mode can determine the likelihood of disease emergence. Indirect transmission routes (such as food‐borne, faecal‐borne or vector‐borne), where hosts do not need to come into direct contact with each other, may increase opportunities for cross‐species exposure and transmission (Woolhouse, Haydon & Antia 2005). In contrast, direct transmission (such as sexual and vertical transmission) characteristically occurs within, rather than between, host species. Viral infections within *A. mellifera* have been well studied and evidence suggests transmission can occur both directly and indirectly (Table S3).

##### Indirect transmission: flower sharing and vectors

Viruses have been detected in a variety of food resources (e.g. pollen, honey, royal jelly) (Shen *et al*. 2005; Chen, Evans & Feldlaufer 2006; Singh *et al*. 2010) as well as in the gut and faeces (Hung 2000; Chen *et al*. 2006; Ribière *et al*. 2007), providing evidence for faecal–oral transmission within *A. mellifera* colonies (Table S3). Most insect pollinators are generalist flower visitors (Waser *et al*. 1996), and flower sharing provides a route for cross‐species transmission by faecal–oral transmission, as has been experimentally shown for the gut parasite *Crithidia bombi* in bumblebees (Durrer & Schmid‐Hempel 1994). IAPV was demonstrated to pass from infected bumblebees to uninfected honeybees and vice versa in a controlled greenhouse experiment, with shared flowers as the only source of contact (Singh *et al*. 2010).

For successful transmission via flower sharing, infected faeces must first be deposited on the flower and then remain viable until acquired by a new host. Floral morphology will influence the likelihood of infected faeces being deposited on flowers, while floral traits such as antimicrobial volatiles, compounds in pollen and nectar, and exposure of flower surfaces to ultraviolet radiation, could influence virus viability and survival time (McArt *et al*. 2014). Additionally, pollinator behaviour will influence virus transmission. Social pollinators can learn to recognize flower resources from conspecifics and heterospecifics and are attracted to flowers by the presence of other pollinators (Dawson & Chittka 2012). Conversely, virus presence may alter floral traits causing pollinators to avoid contaminated flowers (McArt *et al*. 2014) as was the case for flowers experimentally inoculated with *C. bombi* (Fouks & Lattorff 2011).

Vector‐borne transmission is a frequent source of zoonoses. In *A. mellifera*, the vector *V. destructor* has played an important role for viral disease emergence (e.g. Martin *et al*. 2012), but is not directly relevant for cross‐species transmission beyond honeybees, as it is specific to *Apis*. Bumblebees are associated with several phoretic and tracheal mite species. Little is known about their biology or impact on bumblebee populations. However, Schwarz & Huck (1997) found that four species of phoretic mite could actively transfer between flowers and foraging bumblebees, raising the possibility that these mites could spread pathogens. Whether tracheal or phoretic mites of non‐*Apis* pollinators contribute to inter‐ and intraspecific viral transmission are currently unknown and warrants future research. Similarly, it is conceivable that conopid flies, parasitoid diptera that lay their eggs predominantly in adult aculeate hymenoptera, could contribute to disease transmission. Some of these species are known to locally parasitise multiple bumblebee species (Schmid‐Hempel & Schmid‐Hempel 1996), but their potential role in disease transmission has so far remained unexplored.

##### Direct transmission: social parasitism and predation

Social pollinators suffer from a range of social parasites and predatory behaviours that promote direct inter‐ and intraspecific pathogen transmission. For example, both wasps and bumblebees are known to rob honeybee nests; Genersch *et al*. (2006) discovered DWV in a wild *B. pascuorum* colony that was observed robbing honey from nearby DWV‐positive honeybee colonies. Further, in a recent survey, only those wasp (*Vespula vulgaris)* and bumblebee species (*B. terrestris* and *B. pascuorum*) known to rob honeybee colonies were positive for DWV (Evison *et al*. 2012). However, sample sizes were too low to confirm virus absence in the nonrobbing species.

Pollinator colonies are also a valuable resource for social and larval parasitism, which has the potential to lead to disease transmission between the parasite and its host and vice versa. In bumblebees, cuckoo bees from the *Psithyrus* subgenus are obligate parasites, where the female cuckoo bee enters a bumblebee nest, kills the queen and lays eggs that are reared by the social bumblebee workers. Additionally, the larvae of several hoverfly species scavenge in social insect nests, for example *Volucella zonaria* (social wasps, Sommaggio 1999) and *V. pellucens* (social bees and wasps, Coe 1953).

Social bumblebees may also engage in some level of social parasitism that could lead to intraspecific transmission: dubbed ‘egg dumping’, there is microsatellite‐based evidence that conspecific queens may lay eggs in foreign nests (O'Connor, Park & Goulson 2013). Additionally, direct transmission can occur where adult workers ‘drift’, that is when they enter an unrelated nest of the same species. This is a common phenomenon in *A. mellifera* (e.g. Chapman, Beekman & Oldroyd 2010) and has also been experimentally documented in artificial bumblebee colonies (Birmingham *et al*. 2004; Lopez‐Vaamonde *et al*. 2004). While this behaviour is rare in natural bumblebee colonies (O'Connor, Park & Goulson 2013), direct transmission via drifting could be highly relevant where artificial bumblebee colonies are used in close proximity to each other for pollination services.

### Anthropogenic drivers: spillover between managed and wild pollinators

#### Poor husbandry and management

The husbandry techniques used in commercial pollination have potential to increase pathogen exposure. In bumblebees, for example, the cause of the symptomatic DWV infection in *B. terrestris* reported in Genersch *et al*. (2006) was assumed to be the once common practice of housing honeybee workers with bumblebee queens in commercial breeding facilities to encourage the queens to nest. Besides the increased potential for transmission by rearing large numbers of individuals in close proximity, virus‐contaminated pollen (e.g. Singh *et al*. 2010) is a risk to commercial pollinators. Pollen is an essential protein and vitamin source that cannot readily be substituted. In captivity, both bumblebees and honeybees are often fed with pollen collected through traps attached to honeybee colonies, and a number of studies have suggested that feeding untreated virus‐contaminated pollen can result in infected individuals and colonies (Singh *et al*. 2010; Graystock *et al*. 2013).

Unsurprisingly then, studies have found that several pathogens are more prevalent in commercial than wild bumblebee populations (Colla *et al*. 2006; Goka, Okabe & Yoneda 2006). Despite existing regulations and the commitment of commercial breeders to produce pathogen‐free colonies (Meeus *et al*. 2011), a recent molecular study detected five pathogens (DWV, *Nosema bombi*,* N. ceranae*,* C. bombi* and *Apicystis bombi*) across 77% of 48 commercially produced bumblebee colonies (Graystock *et al*. 2013). This agrees with Murray *et al*. (2013), who found that 73·5% of 68 commercial *B. terrestris* colonies were infected either with *Crithidia spp*., *N. bombi*, or both.

Accidental release of infected commercial bumblebees from agricultural systems poses a real risk of transmission to wild pollinators. First, local commercial bumblebee populations can be large: Colla *et al*. (2006) estimated that up to 23 000 bumblebees may pollinate a greenhouse. Secondly, bumblebees regularly escape and forage on noncommercial flower resources. Murray *et al*. (2013) found that pollen collected by commercial *B. terrestris* contained between 31 and 97% noncrop pollen, depending on the agricultural system (i.e. greenhouse, polytunnel and open field), in accordance with a previous study finding 73% noncrop pollen collected by bumblebees released in greenhouses (Whittington *et al*. 2004).

#### Global transportation of commercial species

The globalization of the pollinator industry provides unprecedented opportunities for pathogens to cross geographic and host boundaries. For example, the commercial production of *B. occidentalis* in North America collapsed in the last decade, although direct evidence is lacking (Brown 2011), this has been attributed to infection with the microsporidian *N. bombi,* introduced through commercial European *B. terrestris* colonies in the 1990s. Similarly, *C. bombi* has been found in native bumblebee populations at greenhouse sites where commercial imported colonies were used, but not at control sites (Colla *et al*. 2006). This corresponds with modelled predictions of primary pathogen spillover (Otterstatter & Thomson 2008). Such patterns are not limited to North America: in South America, *C. bombi* and *A. bombi* may have been introduced by the invasive *B. terrestris*, originally imported for greenhouse pollination and now the dominant species across much of Chile and Argentina (Plischuk & Lange 2009; Schmid‐Hempel *et al*. 2014). European haplotypes of the bumblebee tracheal mite *Locustacarus buchneri* were found in commercial *B. ignitus* originally reared in European commercial operations (Goka *et al*. 2001), and later in native Japanese bumble bees, while Japanese haplotypes were found in commercial bees (a ‘spill back’ from wild populations to managed ones) (Goka, Okabe & Yoneda 2006).

#### Global spread of Varroa mite increases prevalence of viruses in Apis host

The most poignant case of disease emergence caused by beekeeping practices is the spread of *Varroa* together with the viruses it promotes. *Varroa* has spread globally since the 1950s after it jumped from its original host *A. cerana* (the Asian honeybee) to *A. mellifera* as a result of the commercial transportation of honeybees (Oldroyd 1999). Much of its pathogenicity is caused by spreading viral diseases and increasing the virulence of otherwise often asymptomatic viral infections, such as DWV (e.g. Martin *et al*. 2012). This increased prevalence may in turn increase transmission to wild pollinators.

## Establishing an infection in the new host

To establish an acute infection, an emerging pathogen has to replicate within its novel host. Pathogen type (Woolhouse, Haydon & Antia 2005) and host relatedness (Davies & Pedersen 2008; Longdon *et al*. 2011) are generally the primary factors determining the range of host species a pathogen can infect. The currently available data suggest common RNA viruses, pathogenic to honeybees, are present in many hosts (Fig. 1). However, most studies have only screened for viral genomes in pollinator field samples using RT‐PCR. Importantly, testing positive for virus presence does not necessarily imply that the pathogen is replicating in its host, but may simply reflect that an individual has ingested viral particles, for example through contaminated pollen. It should be noted, however, that an individual passively carrying a pathogen may still be infectious to others.

Genersch *et al*. (2006) inferred virus replication through identification of DWV symptoms in about 10% of queens in a commercial *B. terrestris* colony. Symptomatic bees were confirmed to be DWV‐positive by RT‐PCR. However, virus symptoms tend to be generic and are rarely diagnostic. For example, *N. bombi* may cause DWV‐like symptoms in *B. terrestris* (Otti & Schmid‐Hempel 2008), while viruses may often persist as asymptomatic infections (Chen & Siede 2007). Other symptomatic viral infections have not been reported in non‐*Apis* pollinators, which may partly be due to biased collection methods: typically in these surveys, foraging pollinators are tested for viral infection (e.g. Singh *et al*. 2010; Evison *et al*. 2012; Levitt *et al*. 2013), so these individuals are capable of flying and are suffering no obvious ill effects. However, sublethal effects have been demonstrated in *B. terrestris* under laboratory conditions, infection with IAPV and KBV reduced worker reproduction (Meeus *et al*. 2014) and DWV reduced mean longevity by 6 days (Fürst *et al*. 2014). In positive‐sense RNA viruses, virus replication can be detected through the specific amplification of the negative‐strand replication intermediate. Several studies have used this diagnostic method across a limited range of host species for DWV, BQCV, IAPV and CBPV, generally finding that RNA viruses replicate in several species, particularly ones closely associated with *A. mellifera* through parasitism or flower sharing (Fig. 1, Table S4).

### Biological drivers: host and parasite genetics

#### The nature of the pathogen

Pathogens vary greatly in host range breadth according to their type. For example, in contrast to the broad host range of ‘honeybee’ RNA viruses, trypanosome gut parasites tend to be more host specific, *that is C. bombi* infecting only bumblebee species, and *C. mellificae* infecting only honeybees (Schmid‐Hempel 1998). RNA viruses have the highest propensity for host shifting (Woolhouse, Haydon & Antia 2005); their high mutation rate, poor mutation‐correction abilities and short replication time allow them to adapt rapidly to new host environments. The accumulation of various mutated viruses, called viral quasi‐species (Domingo & Holland 1997), further increases the probability of successful adaptation to a new host. Viruses use cell receptors to enter host cells and these cell receptors are often conserved across host species, making them susceptible to infection (Woolhouse, Haydon & Antia 2005). For example, the broad host range of the foot‐and‐mouth disease virus (FMDV, a picorna‐like virus related to common pollinator viruses) may be due to the use of conserved receptors (Baranowski, Ruiz‐Jarabo & Domingo 2001). In addition, RNA viruses can often adapt to use novel receptors through few point mutations on the viral capsid, for example, a single amino acid substitution enabled FMDV to infect a new host, the guinea pig (Núñez *et al*. 2001). Identifying conserved receptors and virus mutations may allow for better predictions of the potential host range of viral diseases.

#### Relatedness of hosts

Pathogens are more likely to infect closely related hosts due to their shared evolutionary history (Engelstädter & Hurst 2006). This assumption has been experimentally documented primarily using Drosophila pathogens (Perlman & Jaenike 2003; Engelstädter & Hurst 2006; Longdon *et al*. 2011). Longdon *et al*. (2011) found that host relatedness was the main factor determining a virus’ ability to persist and replicate in a host in the Drosophila–sigma virus system. This suggests that viruses are generally less well adapted to novel cellular environments or immune defence systems of distantly related hosts, even though they may jump phylogenetic divides to cause emerging diseases.

The host range of ‘honeybee’ RNA viruses reported so far includes closely related and phylogenetically diverse species (Fig. 1). While there are no studies systematically comparing infection spread, DWV seems to have a particularly broad host range, with replication detected in several bumblebee species and wasps as well as *V. destructor* and arthropods associated with honeybee hives, whereas other viral infections seem to have a more phylogenetically limited distribution (Fig. 1, Table S4). For DWV, Levitt *et al*. (2013) (USA) and Fürst *et al*. (2014) (UK) found that viral isolates were circulating amongst a range of species. While data are not conclusive at the moment, this suggests that disease emergence in pollinator communities is facilitated by consisting of many closely related species, but is not limited to, for example the social Hymenoptera.

### Anthropogenic factors: Immunosuppression through environmental stress

It may be costly for an insect to mount an immune response against pathogens (Moret & Schmid‐Hempel 2000). Immunosuppression through environmental stressors could increase the risk of infection and lower the threshold for disease emergence. Such stressors can include malnutrition caused by a lack of pollen sources and pollen diversity in areas under intense agricultural use, the use of chemical plant protection agents and the presence of *Varroa*, which can affect the honeybee's immune system (Yang & Cox‐Foster 2005) leading to increased susceptibility to viruses (Vanbergen *et al*. 2013). Pollinators, and especially honeybees, can be exposed to a high level of diverse chemicals (Mullin *et al*. 2010). Neonicotinoid pesticides, for example, three of which are currently under a 2‐year moratorium restricting their use in the EU, can increase susceptibility to DWV infections (Di Prisco *et al*. 2013) by affecting the immune system. Beyond the relatively well‐understood threat of pesticides, the full breadth of chemicals to which pollinators may be exposed needs to be considered. For example, it has become clear that an intact gut microbiome is essential for pathogen resistance (Koch & Schmid‐Hempel 2011), which could be disrupted by chemicals with an antibiotic function.

## Transmission within a new host species

Once the pathogen has established an infection in a novel host individual, its ability to transmit within the novel host population (either in isolation or as a multihost pathogen) will determine whether this remains an isolated spillover event or results in an emerging disease. In other words, it depends on each new infected host individual infecting, on average, more than one individual in its population (i.e. the basic reproductive number *R*_0_ is >1) (Woolhouse, Haydon & Antia 2005). The data currently available are not sufficient to test whether infections in pollinator species represent transient spillovers or if they are part of a sustained transmission cycle. Neither are they sufficient to determine directionality of cross‐species transmission. However, the evolutionary ecology of pollinators as well as management practices may increase the risk of spillovers leading to disease emergence (Fürst *et al*. 2014).

### Biological drivers: sociality

Pollinator species span a gradient of sociality, ranging from solitary species (such as solitary bees or hoverflies), through primitively eusocial species that live in annual colonies of a few hundred individuals (bumblebees and social wasps) to the eusocial honeybees. Although social living brings with it fitness benefits such as cooperative brood care, efficient foraging, mass defence and social immunity (reviewed by Cremer, Armitage & Schmid‐Hempel 2007), it also provides an ideal environment for intraspecific pathogen transmission.

#### Host genetics

Social Hymenoptera live in large, crowded colonies of closely related haplo–diploid individuals. In bumblebees, it has been demonstrated experimentally that parasite transmission is higher between genetically homogeneous individuals (Shykoff & Schmid‐Hempel 1991) and that genetically diverse colonies have decreased parasite loads and higher reproductive success (Baer & Schmid‐Hempel 1999). While multiple mating may increase the risk of venereal disease, it reduces disease burden in *A. mellifera* (Seeley & Tarpy 2006), which is naturally promiscuous, unlike most bumblebee species (Schmid‐Hempel & Crozier 1999).

#### Direct and indirect transmission routes via host social behaviours

Social behaviours such as trophallaxis (exchanging food among colony members), brood care, grooming and hygienic removal of diseased individuals, can increase the potential for disease transmission by faecal‐oral or direct contact routes (Cremer, Armitage & Schmid‐Hempel 2007). Disease transmission may also increase with individual and colony life span, ranging from a few months in bumblebees to years in honeybees. For example, infection intensities of the multihost pathogen *N. bombi* reach a higher level in *B. terrestris* than in *B. lucorum*, the latter of which has a shorter life cycle and smaller colonies (Rutrecht & Brown 2009). Sexual transmission, as demonstrated for DWV in honeybees (de Miranda & Fries 2008), may play a particular role in rapidly spreading pathogens at a landscape scale. In bumblebees, the queens and males show dispersal ranges of several kilometres (Lepais *et al*. 2010), while workers’ foraging trips are typically less than 300 m.

In solitary pollinators, pathogens face different challenges for transmission. In hoverflies, for example, brood care is absent and generations occupy separate niche space, with larvae of different species being carnivorous, phytophagous or scavengers. They inhabit various environments from tree holes to foul water (Branquart & Hemptinne 2000), where they may in turn acquire a different pathogen range. Adults meet only to mate and while feeding on flowers, severely limiting the opportunities for disease transmission as compared to social insects.

### Anthropogenic factors: The use of managed pollinators

High densities within breeding facilities and in commercial pollination operations increase the contact rate between infected and uninfected conspecifics, thereby lowering the threshold for disease emergence. A particular issue in managed populations is the potential for transmission between genetically diverse hosts which could lead to the evolution of general transmission strategies and higher virulence, as has been demonstrated in fish farms (Pulkkinen *et al*. 2010).

### Conclusions

There is potential for cross‐species transmission of RNA viruses in pollinators world‐wide. Exposure does not appear to be a limiting step for virus emergence in pollinators, with current data suggesting that virus spillover events across a broad range of closely and distantly related host species have occurred multiple times in different parts of the world (Fig. 1, Table S4). However, it is still unclear to what degree viruses can then replicate in novel hosts (Fig. 1). Whether these viruses then are able to spread within the new host population has so far not been addressed. Based on identified risk factors (Fig. 2), we propose that the risk of establishing viral transmission within social pollinator populations is higher than for solitary species as their life history and relatedness should lower the threshold for disease emergence.

To assess the threat and impact of disease emergence in pollinator populations, we have to fill a number of key knowledge gaps (Table 1). Fundamentally, we need to understand the true spread of viral diseases in pollinators, how they are transmitted and maintained and what harm they cause. While there is clearly a need for much additional research, it is equally clear that the risk of disease emergence increases with every opportunity for pathogen host switching. Given the key role of pollinators in agriculture and the natural environment, it is evident that we need to minimize the risk of spillover events to mitigate disease emergence.

**Table 1:** Gaps in our knowledge of viral diseases of pollinating insects and future research

Knowledge gaps	Further research
Prevalence and infection outside the *Apis*‐genus	Field studies across a broad taxonomic and geographic range verifying both viral presence and infection status
Viral life cycle ☐ ‐Transmission routes ☐ ‐Transmission through hibernation	Experimental infection studies both in *Apis* and novel hosts. Genetic studies to confirm results in nature
Virulence ☐ ‐Pathogenicity ☐ ‐Effect of host‐switching on virulence	Field and experimental studies to identify lethal and sublethal pathogenic effects across species
Disease emergence ☐ ‐Which viruses have successfully emerged as novel diseases? ☐ ‐What are the epidemiological dynamics in multihost systems? ☐ ‐Is it the cause of pollinator declines?	Field and experimental studies to determine whether transmission is maintained within species and whether there are source/sink dynamics in natural multihost systems

There are many inherent biological factors that increase the risk of disease emergence in pollinator species (Fig. 2), but anthropogenic drivers may be equally important in this system. Crucially, it is these drivers that can be changed by policy and management. Currently, intensively bred alien and/or native species are repeatedly introduced in large numbers, with potentially high pathogen loads, transported globally and released into an environment where they can often freely interact with native populations of related species. Correlative and circumstantial evidence strongly suggests that pathogen spillover from commercial species has occurred to the detriment of native populations (Colla *et al*. 2006; Goka, Okabe & Yoneda 2006; Plischuk & Lange 2009; Cameron *et al*. 2011; Szabo *et al*. 2012), even though there is no direct evidence to date that spillovers have caused epidemics or declines in wild populations (Meeus *et al*. 2011).

To address this risk, first, viruses need to be managed and better monitored in apiculture. If *A. mellifera* acts as a reservoir host for spillover into the pollinator community, then disease control and monitoring in managed populations is essential not only for honeybee health, but for the sake of the wider pollinator community (Fürst *et al*. 2014). It is necessary to routinely screen for pathogens, including viruses, prior to movement across countries (i.e. migratory honeybees in the USA) as well as imports and exports. The *Varroa* mite appears to be the main cause of virus spread throughout *A. mellifera* populations, which by increasing viral prevalence, virulence and geographic range may indirectly affect virus spillover into non‐*Apis* hosts. Thus, controlling the *Varroa* mite and keeping them out of currently *Varroa*‐free areas is essential.

Secondly, the commercial use of bumblebees needs to be more tightly managed. Despite tightened regulations and mandatory screening in some countries, two recent studies worryingly report that over 70% of ‘pathogen‐free’ commercially produced bumblebees were carrying pathogens (Graystock *et al*. 2013; Murray *et al*. 2013). Additionally, Graystock *et al*. (2013) found that the pollen supplied to feed these colonies was also carrying pathogens, including DWV. Irradiating pollen prior to use (Singh *et al*. 2010) and avoiding using honeybee workers to encourage egg laying in captive queens are easy and necessary precautions. While it may not be practical or economically feasible to keep breeding facilities entirely pathogen‐free, routine checks to ensure breeding facilities are not introducing known or novel pathogens and/or strains into wild populations are necessary. The next best policy for elimination is to prevent the escape of commercially bred individuals into the wild by implementing biosecurity measures (Goka 2010). In addition, to prevent introducing invasive pathogens, native pollinator species should be used for commercial pollination and bred locally whenever possible.

Environmental stressors, such as pesticides and habitat degradation, are anthropogenic factors that may potentially increase disease emergence through immunosuppression. Thus, minimizing the exposure to chemicals such as pesticides or acaricides through integrated pest management (Smith & Smith 1949), which aims to balance the need for pest control with minimal pesticide use, is crucial. It is also critical to prevent malnourishment of individuals and colonies, which can be achieved by providing varied floral resources throughout the pollinator season through large‐scale land management. This is an issue the public can be directly involved in: already, gardens provide prime floral resources for pollinators in temperate regions such as the UK (e.g. Goulson *et al*. 2002). By providing such resources and nesting opportunities, the public cannot only bolster pollinator populations themselves but potentially help prevent disease emergence.

## Data accessibility

This review contains no new data. See Supporting Information for references and data used in this review.

## Supporting information


**Table S1.** The global presence and prevalence of eight common viruses of *Apis mellifera*.

**Table S2.** A list of sequenced viruses isolated from *Apis mellifera* to date.

**Table S3.** Transmission routes of eight common viruses of *Apis mellifera*.

**Table S4.** Data on host range for eight common viruses of *Apis mellifera*.
