# Supplementary material for: REVIEW: Emerging viral disease risk to pollinating insects: ecological, evolutionary and anthropogenic factors
Source: J Appl Ecol. 2015 Jan 16;52(2):331–40. doi: 10.1111/1365-2664.12385 (PMC4415536; doi:10.1111/1365-2664.12385)
Supplement: Supplementary file 1 — Table S1. The global presence and prevalence of eight common viruses of Apis mellifera. [file JPE-52-331-s001.pdf]

Table S1.The global presence and estimated prevalence of the eight most common viruses of *Apis mellifera,* plus conditions that trigger pathogenicity and where they've been implicated in recent serious colony losses. Virus abbreviations: DWV - Deformed wing virus, SBV - Sacbrood virus, BQCV - Black queen cell virus, KBV - Kashmir bee virus, ABPV - acute bee paralysis virus, IAPV - Israeli acute paralysis virus, SBPV - Slow bee paralysis virus, CBPV - Chronic bee paralysis virus. Prevalence was estimated using data collected from the following surveys: (Tentcheva *et al.* 2004; Berényi *et al.* 2006; Chen *et al.* 2006; Gauthier *et al.* 2007; Ward *et al.* 2007; Nielson, Nicolaisen & Kryger 2008; Welch *et al.* 2009; Carreck, Ball & Martin 2010; Genersch *et al.* 2010; Singh *et al.* 2010; Runckel *et al.* 2011) (High prevalence +++, medium prevalence ++ and low prevalence +).
*An expanded survey could not confirm this result (vanEngelsdorp *et al.* 2009)

| Virus | Global presence | % prevalence estimate | Pathogenicity association | Colony losses |
|---|---|---|---|---|
| **DWV** | Every continent except Oceania | >80% (+++) | *Varroa* (Bowen-Walker, Martin & Gunn 1999) | Germany (Genersch *et al.* 2010); UK (Carreck, Ball & Martin 2010) |
| **SBV** | Every continent | 10-90% (++) | Seasonal, brood density | |
| **BQCV** | Every continent | >80% (+++) | *Nosema* (Bailey 1982) | |
| **KBV** | N. America, Oceania and Europe | <20% (++) | *Varroa* (Chen *et al.* 2004) | |
| **ABPV** | Every continent except Oceania | High, variable (++) | *Varroa* (Genersch *et al.* 2010) | Germany (Genersch *et al.* 2010) |
| **IAPV** | N. America, Oceania and Europe | <25% in Europe, high elsewhere (++) | *Varroa* (Di Prisco *et al.* 2011) | USA (Cox-Foster 2007)* |
| **SBPV** | Europe and Oceania | <2% (+) | *Varroa* (Carreck, Ball & Martin 2010) | UK (Carreck, Ball & Martin 2010) |
| **CBPV** | Every continent except S. America | <10% (+) | Colony Density (Ribière, Olivier & Blanchard 2010) | |

**References**


Bailey, L. (1982) Viruses of honeybees. *Bee World,* **63,** 165-173.

Berényi, O., Bakonyi, T., Derakhshifar, I., Koglberger, H. & Nowotny, N. (2006) Occurrence of six honeybee viruses in diseased Austrian apiaries. *Applied and Environmental Microbiology,* **72,** 2414-2420.

Bowen-Walker, P.L., Martin, S.J. & Gunn, A. (1999) The transmission of deformed wing virus between honeybees (*Apis mellifera* L.) by the ectoparasitic mite *Varroa jacobsoni* Oud. *Journal of Invertebrate Pathology,* **73,** 101-106.

Carreck, N.L., Ball, B.V. & Martin, S.J. (2010) Honey bee colony collapse and changes in viral prevalence associated with *Varroa destructor*. *Journal of Apicultural Research,* **49,** 93-94.

Chen, Y., Pettis, J., Collins, A. & Feldlaufer, M. (2006) Prevalance and transmission of honeybee viruses. *Applied and Environmental Microbiology,* **72,** 606-611.

Chen, Y., Pettis, J.S., Evans, J.D., Kramer, M. & Feldlaufer, M. (2004) Transmission of Kashmir bee virus by the ectoparasitic mite *Varroa destructor*. *Apidologie,* **35,** 441-448.

Cox-Foster, D.L. (2007) A metagenomic survey of microbes in honey bee colony collapse disorder. *Science,* **318,** 283-287.

Di Prisco, G., Pennacchio, F., Caprio, E., Boncristiani Jr, H.F., Evans, J. & Chen, Y. (2011) *Varroa destructor* is an effective vector of Israeli acute paralysis virus is the honeybee, *Apis mellifera*. *Journal of General Virology,* **92,** 151-155.

Gauthier, L., Tentcheva, D., Tournaire, M., Dainat, B., Cousserans, F., Colin, M.E. & Bergoin, M. (2007) Viral load estimation in asymptomatic honey bee colonies using the quantitative RT-PCR technique. *Apidologie,* **38,** 426-435.

Genersch, E., von der Ohe, W., Kaatz, H., Schroeder, A., Otten, C., Büchler, R., Berg, S., Ritter, W., Mühlen, W., Gisder, S., Meixner, M., Liebig, G. & Rosenkranz, P. (2010) The German bee monitoring project: a long term study to understand periodically high winter losses of honey bee colonies. *Apidologie,* **41,** 332-352.

Nielson, S.L., Nicolaisen, M. & Kryger, P. (2008) Incidence of Acute bee paralysis virus, Black queen cell virus, Chronic bee paralysis virus, Deformed wing virus, Kashmir bee virus and sacbrood virus in honey bees (*Apis mellifera*) in Denmark. *Apidologie,* **39,** 310-314.

Ribière, M., Olivier, V. & Blanchard, P. (2010) Chronic bee paralysis: a disease and a virus like no other? *Journal of Invertebrate Pathology,* **103,** S120-S131.

Runckel, C., Flenniken, M.L., Engel, J.C., Ruby, J.G., Ganem, D., Andino, R. & DeRisi, J.L. (2011) Temporal analysis of the honey bee microbiome reveals four novel viruses and seasonal prevalence of known viruses, *Nosema,* and *Crithidia*. *PLoS ONE,* **6,** e20656.

Singh, R., Levitt, A.L., Rajotte, E.G., Holmes, E.C., Ostiguy, N., vanEngelsdorp, S., Lipkin, W.I., dePamphillis, C.W., Toth, A.L. & Cox-Foster, D.L. (2010) RNA viruses in hymenopteran pollinators: Evidence of inter-taxa virus transmission via pollen and potential impact on non-*Apis* hymenopteran species. *PLoS ONE,* **5,** e14357.

Tentcheva, D., Gauthier, L., Zappulla, N., Dainat, B., Cousserans, F., Colin, M.E. & Bergoin, M. (2004) Prevalence and seasonal variations of six bee viruses in *Apis mellifera* L. and *Varroa destructor* mite populations in France. *Applied and Environmental Microbiology,* **70,** 7185-7191.

vanEngelsdorp, D., Evans, D.J., Saegerman, C., Mullin, C., Haubruge, E., Nguyen, B.K., Frazier, m., Frazier, J., Cox-Foster, D.L., Chen, Y., Underwood, R.M., Tarpy, D.R. & Pettis, J. (2009) Colony collapse disorder: A descriptive study. *PLoS ONE,* **4,** e6481.

Ward, L., Waite, R., Boonham, N., Fisher, T., Pescod, K., Thompson, H., Chantawannakul, P. & Brown, M. (2007) First detection of Kashmir bee virus in the UK using real-time PCR. *Apidologie,* **38,** 181-190.

Welch, A., Drummond, F., Tewari, S., Averill, A. & Burand, J.P. (2009) Presence and prevalence of viruses in local and migratory honeybees (*Apis mellifera*) in Massachusetts. *Applied and Environmental Microbiology,* **75,** 7862-7865.
