# Supplementary material for: REVIEW: Emerging viral disease risk to pollinating insects: ecological, evolutionary and anthropogenic factors
Source: J Appl Ecol. 2015 Jan 16;52(2):331–40. doi: 10.1111/1365-2664.12385 (PMC4415536; doi:10.1111/1365-2664.12385)
Supplement: Supplementary file 2 — Table S2. A list of sequenced viruses isolated from Apis mellifera to date. [file JPE-52-331-s002.pdf]

Table S2. A list of the viruses isolated from *Apis mellifera* that have been sequenced to date (all positive single-stranded RNA genotype), their families, and references. Note: several viruses are so closely related that it is yet to be determined if they are actually members of same species e.g. DWV/VDV-1/KV; ABPV/KBV/IAPV; and LSV-1/LSV-2.

Virus	Abbr.	Family	References
Deformed Wing Virus	DWV	Iflaviridae	de Miranda and Genersch (2010)
Kakugo Virus	KV	Iflaviridae	Fujiyuki *et al.* (2004)
Sacbrood Virus	SBV	Iflaviridae	Bailey and Fernando (1972), Ghosh *et al.* (1999)
Varroa Destructor Virus-1	VDV-1	Iflaviridae	Ongus *et al.* (2004)
Slow Bee Paralysis Virus	SBPV	Iflaviridae	Bailey and Woods (1974), de Miranda *et al.* (2010)
Acute Bee Paralysis Virus	ABPV	Dicistroviridae	Bailey, Gibbs and Woods (1963), de Miranda, Cordoni and Budge (2010)
Israeli Acute Paralysis Virus	IAPV	Dicistroviridae	Maori *et al.* (2007)
Kashmir Bee Virus	KBV	Dicistroviridae	Bailey and Woods (1977), de Miranda *et al.* (2004)
Aphid Lethal Paralysis virus	ALPV	Dicistroviridae	Runckel *et al.* (2011)
Big Sioux River virus	BSRV	Dicistroviridae	Runckel *et al.* (2011)
Black Queen Cell Virus	BQCV	Dicistroviridae	Bailey and Woods (1977), Leat *et al.* (2000)
Chronic Bee Paralysis Virus	CBPV	Unassigned, related to Nodaviridae and Tombusviridae	Bailey, Gibbs and Woods (1963), Olivier *et al.* (2008), Ribière, Olivier and Blanchard (2010)
Lake Sinai Virus 1 & 2	LSV	Unassigned, related to Nodaviridae and Tombusviridae	Runckel *et al.* (2011)
Tobacco Ring Spot Virus	TRSV	Comoviridae	Li *et al.* (2014)

References

Bailey, L. & Fernando, E.F. (1972) Effects of Sacbrood Virus on adult honey bees. *Annals of Applied Biology,* **72,** 27-&.

Bailey, L., Gibbs, A.J. & Woods, R.D. (1963) Two viruses from adult honey bees (*Apis mellifera* Linnaeus). *Virology* **21,** 390-395.

Bailey, L. & Woods, R.D. (1974) Three previously undescribed viruses from the honey bee. *Journal of General Virology,* **25,** 175-186.

Bailey, L. & Woods, R.D. (1977) 2 more small RNA viruses from honey bees and further observations on Sacbrood and Acute Bee Paralysis Viruses. *Journal of General Virology,* **37,** 175-182.

de Miranda, J.R., Cordoni, G. & Budge, G. (2010) The Acute bee paralysis virus-Kashmir bee virus-Israeli acute paralysis virus complex. *Journal of Invertebrate Pathology,* **103,** S30-S47.

de Miranda, J.R., Dainat, B., Locke, B., Cordoni, G., Berthoud, H., Gauthier, L., Neumann, P., Budge, G.E., Ball, B.V. & Stoltz, D.B. (2010) Genetic characterization of Slow Bee Paralysis Virus of the honeybee (*Apis mellifera* L.). *Journal of General Virology,* **91,** 2524-2530.

de Miranda, J.R., Drebot, M., Tyler, S., Shen, M., Cameron, C.E., Stoltz, D.B. & Camazine, S.M. (2004) Complete nucleotide sequence of Kashmir bee virus and comparison with acute bee paralysis virus. *Journal of General Virology,* **85,** 2263-2270.

de Miranda, J.R. & Genersch, E. (2010) Deformed Wing Virus. *Journal of Invertebrate Pathology,* **103,** S48-S61.

Fujiyuki, T., Takeuchi, H., Ono, M., Ohka, S., Sasaki, T., Nomoto, A. & Kubo, T. (2004) Novel insect picorna-like virus identified in the brains of aggressive worker honeybees. *Journal of Virology,* **78,** 1093-1100.

Ghosh, R.C., Ball, B.V., Willcocks, M.M. & Carter, M.J. (1999) The nucleotide sequence of Sacbrood Virus of the honey bee: an insect picorna-like virus. *Journal of General Virology,* **80,** 1541-1549.

Leat, N., Ball, B., Govan, V. & Davison, S. (2000) Analysis of the complete genome sequence of Black Queen Cell Virus, a picorna-like virus of honey bees. *Journal of General Virology,* **81,** 2111-2119.

Li, J.L., Cornman, R.S., Evans, J.D., Pettis, J.S., Zhao, Y., Murphy, C., Peng, W.J., Wu, J., Hamilton, M., Boncristiani, H.F.J., Zhou, L., Hammond, J. & Chen, Y.P. (2014) Systemic spread and propagation of a plant-pathogenic virus in European honeybees, *Apis mellifera*. *mBio,* **5,** e00898-00813.

Maori, E., Lavi, S., Mozes-Koch, R., Gantman, Y., Peretz, Y., Edelbaum, O., Tanne, E. & Sela, I. (2007) Isolation and characterization of Israeli acute paralysis virus, a discistrovirus affecting honeybees in Israel: evidence for diversity due to intra- and inter-species recombination. *Journal of General Virology,* **88,** 3428-3438.

Olivier, V., Blanchard, P., Chaouch, S., Lallemand, P., Schurr, F., Celle, O., Dubois, E., Tordo, N., Thiery, R., Houlgatte, R. & Ribiere, M. (2008) Molecular characterisation and phylogenetic analysis of Chronic bee paralysis virus, a honey bee virus. *Virus Research,* **132,** 59-68.

Ongus, J.R., Peters, D., Bonmatin, J., Bengsch, E., Vlak, J.M. & van Oers, M.M. (2004) Complete sequence of a picorna-like virus of the genus *Iflavirus* replicating in the mite *Varroa destructor*. *Journal of General Virology,* **85,** 3747-3755.

Ribière, M., Olivier, V. & Blanchard, P. (2010) Chronic bee paralysis: a disease and a virus like no other? *Journal of Invertebrate Pathology,* **103,** S120-S131.

Runckel, C., Flenniken, M.L., Engel, J.C., Ruby, J.G., Ganem, D., Andino, R. & DeRisi, J.L. (2011) Temporal analysis of the honey bee microbiome reveals four novel viruses and seasonal prevalence of known viruses, *Nosema,* and *Crithidia*. *PLoS ONE,* **6,** e20656.
