# Supplementary material for: REVIEW: Emerging viral disease risk to pollinating insects: ecological, evolutionary and anthropogenic factors
Source: J Appl Ecol. 2015 Jan 16;52(2):331–40. doi: 10.1111/1365-2664.12385 (PMC4415536; doi:10.1111/1365-2664.12385)
Supplement: Supplementary file 3 — Table S3. Transmission routes of eight common viruses of Apis mellifera. [file JPE-52-331-s003.pdf]

Table S3. Routes of transmission for the eight most common RNA viruses of *Apis mellifera* with references. Virus abbreviations: DWV - Deformed wing virus, BQCV - Black queen cell virus, ABPV - Acute bee paralysis virus, SBPV - Slow bee paralysis virus, KBV - Kashmir bee virus, CBPV - Chronic bee paralysis virus, SBV - Sacbrood virus, IAPV - Israeli acute paralysis virus. Key: ✓evidence suggestive, ☑ solid evidence (note: evidence was considered 'solid' if a transmission route was directly tested and proven to occur without the possibility of alternative transmission routes occurring simultaneously; evidence was considered 'suggestive' if transmission was shown indirectly (i.e. virus presence in pollen does not prove faecal-oral transmission occurs), was not the main focus of the study, or alternative routes of transmission were not ruled out).

	Indirect transmission			Direct transmission		
**Viruses**	**Food**	**Faecal**	**Vector (*V.destructor*)**	**Contact**	**Sexual**	**Transovarial**
**DWV**	✓ [1,15]	✓ [10]	☑ [**3**,11,12,14]		✓ [7,16,19]	✓ [10,17,19]
**BQCV**	✓ [1,15]	✓ [10]				✓ [10]
**ABPV**	✓ [1]		✓ [11,18]	✓ [5]		
**SBPV**			☑ [14, **20**]			
**KBV**	✓ [1,2]	✓ [4]	☑ [**2**,9,11]			✓ [10,2]
**CBPV**	✓ [1]	☑ [**6**]		✓ [8]		✓ [10]
**SBV**	✓ [1,2,15]		✓ [11]			✓ [10,2]
**IAPV**			☑ [**13**]			

1. Chen, Evans and Feldlaufer (2006)
2. Shen *et al.* (2005)
3. Bowen-Walker, Martin and Gunn (1999)
4. Hung (2000)
5. Bailey, Gibbs and Woods (1963)
6. Ribière *et al.* (2007)
7. Yañez *et al.* (2012)
8. Bailey, Ball and Perry (1983)
9. Chen *et al.* (2004)
10. Chen *et al.* (2006)
11. Tentcheva *et al.* (2004)
12. Yue and Genersch (2005)
13. Di Prisco *et al.* (2011)
14. Carreck, Ball and Martin (2010)
15. Singh *et al.* (2010)
16. Yue *et al.* (2006)
17. Yue *et al.* (2007)
18. Genersch *et al.* (2010)
19. de Miranda and Fries (2008)
20. Santillán-Galicia *et al.* (2014)

References

Bailey, L., Ball, B. & Perry, J.N. (1983) Honeybee paralysis: Its natural spread and its diminished incidence in England and Wales. *Journal of Apicultural Research,* **22,** 191-195.

Bailey, L., Gibbs, A.J. & Woods, R.D. (1963) Two viruses from adult honey bees (*Apis mellifera* Linnaeus). *Virology* **21,** 390-395.

Bowen-Walker, P.L., Martin, S.J. & Gunn, A. (1999) The transmission of deformed wing virus between honeybees (*Apis mellifera* L.) by the ectoparasitic mite *Varroa jacobsoni* Oud. *Journal of Invertebrate Pathology,* **73,** 101-106.

Carreck, N.L., Ball, B.V. & Martin, S.J. (2010) Honey bee colony collapse and changes in viral prevalence associated with *Varroa destructor*. *Journal of Apicultural Research,* **49,** 93-94.

Chen, Y., Pettis, J., Collins, A. & Feldlaufer, M. (2006) Prevalance and transmission of honeybee viruses. *Applied and Environmental Microbiology,* **72,** 606-611.

Chen, Y., Pettis, J.S., Evans, J.D., Kramer, M. & Feldlaufer, M. (2004) Transmission of Kashmir bee virus by the ectoparasitic mite *Varroa destructor*. *Apidologie,* **35,** 441-448.

Chen, Y.P., Evans, J. & Feldlaufer, M. (2006) Horizontal and vertical transmission of viruses in the honey bee, *Apis mellifera*. *Journal of Invertebrate Pathology,* **92,** 152-159.

de Miranda, J.R. & Fries, I. (2008) Venereal and vertical transmission of Deformed Wing Virus in honeybees (*Apis mellifera* L.). *Journal of Invertebrate Pathology,* **98,** 184-189.

Di Prisco, G., Pennacchio, F., Caprio, E., Boncristiani Jr, H.F., Evans, J. & Chen, Y. (2011) *Varroa destructor* is an effective vector of Israeli acute paralysis virus is the honeybee, *Apis mellifera*. *Journal of General Virology,* **92,** 151-155.

Genersch, E., von der Ohe, W., Kaatz, H., Schroeder, A., Otten, C., Büchler, R., Berg, S., Ritter, W., Mühlen, W., Gisder, S., Meixner, M., Liebig, G. & Rosenkranz, P. (2010) The German bee monitoring project: a long term study to understand periodically high winter losses of honey bee colonies. *Apidologie,* **41,** 332-352.

Hung, A.C.F. (2000) PCR detection of Kashmir bee virus in honey bee excreta. *Journal of Apicultural Research,* **39,** 103-106.

Ribière, M., Lallemand, P., Iscache, A.L., Schurr, F., Celle, O., Blanchard, P., Olivier, V. & Faucon, J.P. (2007) Spread of infectious Chronic bee paralysis virus by honeybee (*Apis mellifera* L.) feces. *Applied and Environmental Microbiology,* **73,** 7711-7716.

Santillán-Galicia, M.T., Ball, B.V., Clark, S.J. & Alderson, P.G. (2014) Slow bee paralysis virus and its transmission in honey bee pupae by Varroa destructor. *Journal of Apicultural Research,* **53,** 146-154.

Shen, M., Cui, L., Ostiguy, N. & Cox-Foster, D.L. (2005) Intricate transmission routes and interactions between picorna-like viruses (Kashmir bee virus and Sacbrood virus) with the honeybee host and the parasitic varroa mite. *Journal of General Virology,* **86,** 2281-2289.

Singh, R., Levitt, A.L., Rajotte, E.G., Holmes, E.C., Ostiguy, N., vanEngelsdorp, S., Lipkin, W.I., dePamphillis, C.W., Toth, A.L. & Cox-Foster, D.L. (2010) RNA viruses in hymenopteran pollinators: Evidence of inter-taxa virus transmission via pollen and potential impact on non-*Apis* hymenopteran species. *PLoS ONE,* **5,** e14357.

Tentcheva, D., Gauthier, L., Zappulla, N., Dainat, B., Cousserans, F., Colin, M.E. & Bergoin, M. (2004) Prevalence and seasonal variations of six bee viruses in *Apis mellifera* L. and *Varroa destructor* mite populations in France. *Applied and Environmental Microbiology,* **70,** 7185-7191.

Yañez, O., Jaffé, R., Jarosch, A., Fries, I., Moritz, R., F,A., Paxton, R.J. & de Miranda, J.R. (2012) Deformed wing virus and drone mating flights in the honey bee (*Apis mellifera*): implications for sexual transmission of a major honey bee virus. *Apidologie,* **43,** 17-30.

Yue, C. & Genersch, E. (2005) RT-PCR analysis of *Deformed wing virus* in honeybees (*Apis mellifera*) and mites (*Varroa destructor*). *Journal of General Virology,* **86,** 3419-3424.

Yue, C., Schröder, M., Bienefeld, K. & Genersch, E. (2006) Detection of viral sequences in semen of honeybees (*Apis mellifera*): evidence for vertical transmission of viruses through drones. *Journal of Invertebrate Pathology,* **92,** 105-108.

Yue, C., Schröder, M., Gisder, S. & Genersch, E. (2007) Vertical-transmission routes for Deformed wing virus of honeybees (*Apis mellifera*). *Journal of General Virology,* **88,** 2329-2336.
