# Supplementary material for: REVIEW: Emerging viral disease risk to pollinating insects: ecological, evolutionary and anthropogenic factors
Source: J Appl Ecol. 2015 Jan 16;52(2):331–40. doi: 10.1111/1365-2664.12385 (PMC4415536; doi:10.1111/1365-2664.12385)
Supplement: Supplementary file 4 — Table S4. Data on host range for eight common viruses of Apis mellifera. [file JPE-52-331-s004.pdf]

Table S4. Virus host range for the eight most common RNA viruses of *Apis mellifera* (virus abbreviations: DWV - Deformed wing virus, BQCV - Black queen cell virus, ABPV - Acute bee paralysis virus, SBPV - Slow bee paralysis virus, SBV - Sacbrood virus, KBV - Kashmir bee virus, IAPV - Israeli acute paralysis virus, CBPV - Chronic bee paralysis virus).

Virus	Host	Location	Sample n	~ % n infected	Replication	Symptomatic	Ref.
DWV	*Bombus terrestris*	Europe commercial	no data	10%	Not tested	Y*	Genersch et al. (2006)
		UK, mainly southwest	57	30%	Not tested	N	Evison et al. (2012)
		Scotland, UK	Unpublished data	Unpublished data	Not tested	N	McMahon pers. comm.
		UK	170	9	Y	N	Fürst et al. (2014)
	Bombus pascuorum	Germany	1 wild nest	No data	Not tested	N	Genersch et al. (2006)
		UK, mainly southwest	36	14%	Not tested	N	Evison et al. (2012)
		UK	60	4	N	N	Fürst et al. (2014)
	Bombus lucorum	Scotland, UK	Unpublished data	Unpublished data	Not tested	N	McMahon pers. comm.
		UK	60	18	Y	N	Fürst et al. (2014)
	Bombus lapidarius	UK	175	16	Y	N	Fürst et al. (2014)
	Bombus huntii	Utah, USA	30	no data	Y (gut only)	N	Li et al. (2011)
	Bombus impatiens	Pennsylvania, USA	5	100%	Not tested	N	Singh et al. (2010)
		Pennsylvania, USA	13	62%	Y	N	Levitt et al. (2013)
	Bombus vagans	Pennsylvania, USA	2	50%	Y	N	Levitt et al. (2013)
	Bombus tenarius	Pennsylvania, USA	2	100%	Not tested	N	Singh et al. (2010)
	Bombus horturum	UK	20	0	n/a	n/a	Fürst et al. (2014)
	Bombus monticola	UK	10	11	Y	N	Fürst et al. (2014)
	Osmia bicornis	Belgium	3 pooled samples (10 per sample)	n/a	Not tested	N	Ravoet et al. (2014)
	Vespula vulgaris	UK, mainly southwest	45	29%	Not tested	N	Evison et al. (2012)
		Pennsylvania, USA	12	92%	Not tested	N	Singh et al. (2010)
	Vespula spp.	Pennsylvania, USA	7	57%	Y	N	Levitt et al. (2013)
	Apis cerana	China (19 provinces)	570	33, 51 and 92% in 3 provinces	Not tested	n/a	Li et al. (2011)
	Apis florae	Xishuang province, China	134	15.6%	Not tested	n/a	Zhang et al. (2012)
	Apis dorsata	Xishuang province, China	190	11.6%	Not tested	n/a	Zhang et al. (2012)
	Blatella germanica	Pennsylvania, USA	8	100%	Y	N	Levitt et al. (2013)
	Aethina tumida	Pennsylvania, USA	21	72%	Not tested	N	Levitt et al. (2013)
	Forficula auricularia	Pennsylvania, USA	10	100%	Y	N	Levitt et al. (2013)
	Galleria mellonella	Pennsylvania, USA	21	71%	N	N	Levitt et al. (2013)
	Araneae (order)	Pennsylvania, USA	10	80%	Not tested	N	Levitt et al. (2013)

	Varroa destructor	UK apiaries (Devon)	462 (3 hives)	No data	Not tested	n/a	Bowen-Walker et al. (1999)
		France apiaries (nationwide)	22,000 (22 apiaries)	100%	Not tested	n/a	Tentcheva et al. (2004)
		France (360 apiaries)	36,000 (36 apiaries)	No data	Not tested	n/a	Gauthier et al. (2007)
		Germany	40 per hive (5 hives)	45-100% per hive	Y (only mites from symptomatic bees)	n/a	Yue and Genersch (2005)
BQCV	*B. huntii*	Utah, USA	30	No data	Y (gut only)	N	Peng et al. (2011)
	B. terrestris	Scotland, UK	Unpublished data	Unpublished data	Not tested	N	McMahon pers. comm.
	B. pascuorum	Scotland, UK	Unpublished data	Unpublished data	Not tested	N	McMahon pers. comm.
	B. lucorum	Scotland, UK	Unpublished data	Unpublished data	Not tested	N	McMahon pers. comm.
	Bombus hortorum	Scotland, UK	Unpublished data	Unpublished data	Not tested	N	McMahon pers. comm.
	B impatiens	Pennsylvania, USA	5	60%	Not tested	N	Singh et al. (2010)
		Pennsylvania, USA	13	61%	Not tested	N	Levitt et al. (2013)
	B. tenarius	Pennsylvania, USA	2	50%	Not tested	N	Singh et al. (2010)
	B. vagans	Pennsylvania, USA	1	100%	Not tested	N	Singh et al. (2010)
		Pennsylvania, USA	2	100%	Not tested	N	Levitt et al. (2013)
	Osmia cornuta	Belgium	3 pooled samples (10 per sample)	n/a	Not tested	N	Ravoet et al. (2014)
	Andrena vaga	Belgium	1 pooled sample (10 in pool)	n/a	Not tested	N	Ravoet et al. (2014)
	Heriades truncorum	Belgium	3 pooled samples (10 per sample)	n/a	Not tested	N	Ravoet et al. (2014)
	A. cerana	China (19 provinces)	570	12% – 98% across 6 provinces	Not tested	N	Li et al. (2012)
	A. florae	Xishuang province, China	134	52%	Not tested	N	Zhang et al. (2012)
	A. dorsata	Xishuang province, China	190	21.6%	Not tested	N	Zhang et al. (2012)
	V. vulgaris	UK, mainly southwest	45	2.2%	Not tested	N	Evison et al. (2012)
		Pennsylvania, USA	12	66.7%	Not tested	N	Singh et al. (2010)
	Vespula sp.	Pennsylvania, USA	7	29%	Not tested	N	Levitt et al. (2013)
	Blatella germanica	Pennsylvania, USA	8	50%	Not tested	N	Levitt et al. (2013)
	Aethina tumida	Pennsylvania, USA	21	5%	Not tested	N	Levitt et al. (2013)
	Forficula auricularia	Pennsylvania, USA	10	60%	Not tested	N	Levitt et al. (2013)
	Galleria mellonella	Pennsylvania, USA	21	33%	Not tested	N	Levitt et al. (2013)
	Araneae (order)	Pennsylvania, USA	10	10%	Not tested	N	Levitt et al. (2013)

ABPV	*B. lucorum*	n/a experimental infections	No data	No data	Not tested	Y	Bailey and Gibbs (1964)
		Scotland, UK	Unpublished data	Unpublished data	Unpublished data	N	Wilfert unpublished
	B. ruderarius	n/a experimental infections	No data	No data	Not tested	Y	Bailey and Gibbs (1964)
	B. terrestris	n/a experimental infections	No data	No data	Not tested	Y	Bailey and Gibbs (1964)
		Scotland, UK	Unpublished data	Unpublished data	Unpublished data	N	Wilfert unpublished
	B. hortorum	n/a experimental infections	No data	No data	Not tested	Y	Bailey and Gibbs (1964)
		Scotland, UK	Unpublished data	Unpublished data	Unpublished data	N	Wilfert unpublished
	B pascuorum	n/a experimental infections	No data	No data	Not tested	N	Bailey and Gibbs (1964)**
		Scotland, UK	Unpublished data	Unpublished data	Unpublished data	N	Wilfert unpublished
	V. destructor	France (nationwide)	22,000 (22 apiaries)	36%	Not tested	n/a	Tentcheva et al. (2004)
		France (360 colonies)	36,000	low	Not tested	n/a	Gauthier et al. (2007)
SBPV	*B. terrestris*	Scotland, UK	Unpublished data	Unpublished data	Unpublished data	N	Wilfert unpublished
	B pascuorum	Scotland, UK	Unpublished data	Unpublished data	Unpublished data	N	Wilfert unpublished
	B. lucorum	Scotland, UK	Unpublished data	Unpublished data	Unpublished data	N	Wilfert unpublished
	B. horturum.	Scotland, UK	Unpublished data	Unpublished data	Unpublished data	N	Wilfert unpublished
	Eristalis pertinax	Scotland, UK	Unpublished data	Unpublished data	Unpublished data	N	Wilfert unpublished
	Episyrphus balteatus	Scotland, UK	Unpublished data	Unpublished data	Unpublished data	N	Wilfert unpublished
SBV	*B. ternarius*	Pennsylvania, USA	2	50%	Not tested	N	Singh et al. (2010)
	B vagans	Pennsylvania, USA	1	100%/	Not tested	N	Singh et al. (2010)
		Pennsylvania, USA	2	50%	Not tested	N	Levitt et al. (2013)
	B. impatiens	Pennsylvania, USA	13	23%	Not tested	N	Levitt et al. (2013)
	V. vulgaris	Pennsylvania, USA	7	57%	Not tested	N	Singh et al. (2010)
		UK, mainly southwest	45	0.45%	Not tested	N	Evison et al. (2012)
	Vespula spp.	Pennsylvania, USA	7	0.07%	Not tested	N	Levitt et al. (2013)
	Blatella germanica	Pennsylvania, USA	8	75%	Not tested		Levitt et al. (2013)
	Aethina tumida	Pennsylvania, USA	21	11%	Not tested	N	Levitt et al. (2013)

	Forficula auricularia	Pennsylvania, USA	10	50%	Not tested	N	Levitt et al. (2013)
	Galleria mellonella	Pennsylvania, USA	21	29%	Not tested	N	Levitt et al. (2013)
	Araneae (order)	Pennsylvania, USA	10	10%	Not tested	N	Levitt et al. (2013)
	V. destructor	Penn State, USA	111 (one hive)	no data	Not tested	n/a	Shen et al. (2005)
		France (nationwide)	22,000 (22 apiaries)	45%	Not tested	n/a	Tentcheva et al. (2004)
KBV	*A. cerana*	India, Kashmir	No data	No data	Not tested	Y	Bailey and Woods (1977)
	Aethina tumida	Pennsylvania, USA	21	44%	Not tested	N	Levitt et al. (2013)
	Forficula auricularia	Pennsylvania, USA	10	20%	Not tested	N	Levitt et al. (2013)
	Galleria mellonella	Pennsylvania, USA	21	29%	Not tested	N	Levitt et al. (2013)
	V. destructor	France (nationwide)	22,000 (22 apiaries)	5%	Not tested	n/a	Tentcheva et al. (2004)
	V. destructor	Penn State, USA	111 (one hive)	No data	Not tested	n/a	Shen et al. (2005)
IAPV	*B. impatiens*	Pennsylvania, USA	13	31%	Y	N	Levitt et al. (2013)
	B. ternarius	Pennsylvania, USA	2	100%	Not tested	N	Singh et al. (2010)
	B. vagans	Pennsylvania, USA	1	100%	Not tested	N	Singh et al. (2010)
	V. vulgaris	Pennsylvania, USA	7	~71%	Not tested	N	Singh et al. (2010)
	Vespa velutina	Hangzhou, China	10	100%	Y (head, thorax & abdomen)	N	Yañez et al. (2012)
	A. cerana	Hangzhou, China	180 (6 hives)	1 hive only	N***	N	Yañez et al. (2012)
	Aethina tumida	Pennsylvania, USA	21	78%	Not tested	N	Levitt et al. (2013)
	Forficula auricularia	Pennsylvania, USA	10	40%	Not tested	N	Levitt et al. (2013)
	Galleria mellonella	Pennsylvania, USA	21	14%	Not tested	N	Levitt et al. (2013)
	Araneae (order)	Pennsylvania, USA	10	10%	Not tested	N	Levitt et al. (2013)
	V. destructor	n/a (experimental)	40	n/a	Y	n/a	Di Prisco et al. (2011)
CBPV	*Camponotus vagus*	France	22 (from 2 apiaries)	No data	Y	N	Celle et al. (2008)
	Formica rufa	France	20 (from 2 apiaries)	No data	N	N	Celle et al. (2008)
	V. destructor	France	19 (from 1 apiary)	No data	Y	n/a	Celle et al. (2008)

* Bumblebees had deformed wings and were found to be positive for DWV by PCR. However, it is not proven that the symptoms are linked to the virus.

** The authors use the now out-of-date name *Bombus agrorum* to refer to B. pascuorum

***The authors do not believe this result to be true as replication occurred in *A. mellifera* and the two species are so similar that it seems unlikely replication does not occur in *A. cerana* as well (Yañez et al. 2012)

References

Bailey, L. and A. J. Gibbs. 1964. Acute infection of bees with paralysis virus. Journal of Insect Pathology **6**:395-407.

Bailey, L. and R. D. Woods. 1977. 2 more small RNA viruses from honey bees and further observations on Sacbrood and Acute Bee Paralysis Viruses. Journal of General Virology **37**:175-182.

Bowen-Walker, P. L., S. J. Martin, and A. Gunn. 1999. The transmission of deformed wing virus between honeybees (*Apis mellifera* L.) by the ectoparasitic mite *Varroa jacobsoni* Oud. Journal of Invertebrate Pathology **73**:101-106.

Celle, O., P. Blanchard, V. Olivier, F. Schurr, N. Cougoule, J. P. Faucon, and M. Ribière. 2008. Detection of Chronic Bee Paralysis Virus (CBPV) genome and its replicative RNA form in various hosts and possible ways of spread. Virus Research **133**:280-284.

Di Prisco, G., F. Pennacchio, E. Caprio, H. F. Boncristiani Jr, J. Evans, and Y. Chen. 2011. *Varroa destructor* is an effective vector of Israeli acute paralysis virus is the honeybee, *Apis mellifera*. Journal of General Virology **92**:151-155.

Evison, S. E. F., K. E. Roberts, L. Laurenson, S. Pietravalle, J. Hui, J. C. Biesmeijer, J. E. Smith, G. Budge, and W. O. H. Hughes. 2012. Pervasiveness of parasites in pollinators. PLoS ONE **7**:e30641.

Fürst, M. A., D. P. McMahon, J. L. Osborne, R. J. Paxton, and M. J. F. Brown. 2014. Disease associations between honeybees and bumblebees as a threat to wild pollinators. Nature **506**:364-366.

Gauthier, L., D. Tentcheva, M. Tournaire, B. Dainat, F. Cousserans, M. E. Colin, and M. Bergoin. 2007. Viral load estimation in asymptomatic honey bee colonies using the quantitative RT-PCR technique. Apidologie **38**:426-435.

Genersch, E., C. Yue, I. Fries, and J. R. De Miranda. 2006. Detection of Deformed Wing Virus, a honey bee viral pathogen, in bumble bees (*Bombus terrestris* and *Bombus pascuorum*) with wing deformities. Journal of Invertebrate Pathology **91**:61-63.

Levitt, A. L., R. Singh, D. L. Cox-Foster, E. Rajotte, K. Hoover, N. Ostiguy, and E. C. Holmes. 2013. Cross-species transmission of honey bee viruses in associated arthropods. Virus Research **176**:232-240.

Li, J., W. Peng, J. Wu, J. P. Strange, H. Boncristiani, and Y. Chen. 2011. Cross-species infection of deformed wing virus poses a new threat to pollinator conservation. Journal of Economic Entomology **104**:732-739.

Li, J., H. Qin, J. Wu, B. M. Sadd, X. Wang, J. Evans, W. Peng, and Y. Chen. 2012. The prevalence of parasites and pathogens in Asian honeybees *Apis cerana* in China. PLoS ONE **7**:e47955.

Peng, W., J. Li, H. F. Boncristiani Jr, J. P. Strange, M. Hamilton, and Y. P. Chen. 2011. Host range expansion of honey bee Black Queen Cell Virus in the bumble bee, *Bombus huntii*. Apidologie **42**:650-658.

Ravoet, J., L. De Smet, I. Meeus, G. Smagghe, T. Wenseleers, and D. C. de Graaf. 2014. Widespread occurence of honey bee pathogens in solitary bees. Journal of Invertebrate Pathology **122**:55-58.

Shen, M., L. Cui, N. Ostiguy, and D. L. Cox-Foster. 2005. Intricate transmission routes and interactions between picorna-like viruses (Kashmir bee virus and Sacbrood virus) with the honeybee host and the parasitic varroa mite. Journal of General Virology **86**:2281-2289.

Singh, R., A. L. Levitt, E. G. Rajotte, E. C. Holmes, N. Ostiguy, S. vanEngelsdorp, W. I. Lipkin, C. W. dePamphillis, A. L. Toth, and D. L. Cox-Foster. 2010. RNA viruses in hymenopteran pollinators: Evidence of inter-taxa virus transmission via pollen and potential impact on non-*Apis* hymenopteran species. PLoS ONE **5**:e14357.

Tentcheva, D., L. Gauthier, N. Zappulla, B. Dainat, F. Cousserans, M. E. Colin, and M. Bergoin. 2004. Prevalence and seasonal variations of six bee viruses in *Apis mellifera* L. and *Varroa destructor* mite populations in France. Applied and Environmental Microbiology **70**:7185-7191.

Yañez, O., H. Zheng, F. Hu, P. Neumann, and V. Dietemann. 2012. A scientific note on Israeli acute paralysis virus infection of Eastern honeybee *Apis cerana* and vespine predator *Vespa velutina*. Apidologie **43**:587-589.

Yue, C. and E. Genersch. 2005. RT-PCR analysis of *Deformed wing virus* in honeybees (*Apis mellifera*) and mites (*Varroa destructor*). Journal of General Virology **86**:3419-3424.

Zhang, X., S. Y. He, J. D. Evans, J. S. Pettis, G. F. Yin, and Y. P. Chen. 2012. New evidence that Deformed wing virus and Black queen cell virus are multi-host pathogens. Journal of Invertebrate Pathology **109**:156-159.
